# Stuck between a rock and a hard place: heart failure with bilateral atrial appendage thrombi and disseminated intravascular coagulation—a case report

**DOI:** 10.1093/ehjcr/ytaf043

**Published:** 2025-02-06

**Authors:** Pietro Mazzeo, Gabriella Bufano, Vincenzo Fioretti, Maria Delia Corbo, Eugenio Stabile

**Affiliations:** Cardiovascular Department, Azienda Ospedaliera Regionale ‘San Carlo’, via Potito Petrone, Potenza 85100, Italy; Cardiovascular Department, Azienda Ospedaliera Regionale ‘San Carlo’, via Potito Petrone, Potenza 85100, Italy; Cardiovascular Department, Azienda Ospedaliera Regionale ‘San Carlo’, via Potito Petrone, Potenza 85100, Italy; Cardiovascular Department, Azienda Ospedaliera Regionale ‘San Carlo’, via Potito Petrone, Potenza 85100, Italy; Cardiovascular Department, Azienda Ospedaliera Regionale ‘San Carlo’, via Potito Petrone, Potenza 85100, Italy

**Keywords:** Case report, Heart failure, RAA thrombosis, Cardiac masses, Atrial thrombosis, Atrial fibrillation, Disseminated intravascular coagulation, Transoesophageal echocardiogram, Positron emission tomography/computed tomography scan

## Abstract

**Background:**

The simultaneous occurrence of left atrial appendage (LAA) and right atrial appendage (RAA) thrombosis is a rare finding in atrial fibrillation (AF). In addition, concomitant conditions, such as heart failure (HF) and disseminated intravascular coagulation (DIC), could be associated with intracardiac thrombosis. *Morganella morganii* is an emerging pathogen, and the association with DIC and cardiac thrombosis is not yet described.

**Case summary:**

A 69-year-old Caucasian man was admitted to the hospital for progressive dyspnoea and new-onset diarrhoea. His physical examination revealed signs of HF and new-onset AF; laboratory tests showed marked thrombocytopaenia and coagulopathy. Blood and urine cultures were positive for *M. morganii*, and the International Society on Thrombosis and Hemostasis criteria were diagnostic for DIC. Transthoracic echocardiogram revealed a large, mobile left atrial mass and severely reduced left ventricular function. Transoesophageal echocardiogram showed two masses: one in the LAA and one in the RAA. Positron emission tomography/computed tomography scan excluded infective endocarditis and malignancies. After the beginning of antibiotic therapy and anticoagulation, the patient developed severe bleeding. Unfortunately, his haemodynamic status was complicated by multi-organ failure, and at the end, he developed irreversible cardiogenic shock. We present this challenging case of HF and AF complicated by DIC in the context of *M. morganii* infection.

**Discussion:**

Multiple mechanisms, such as inflammatory storm, activation of coagulation cascade, and amplified immune response, may explain the hyper-coagulable state related to DIC. In our case, HF, AF, and DIC may have facilitated RAA thrombosis, which is a rare finding. To the best of our knowledge, this is the first reported case of concomitant cardiac thrombosis as a complication of DIC in the context of *M. morganii* infection. This highlights the need among clinicians for an increased awareness about this pathogen and the complications of its infection.

**Learning points**

- Heart failure patients have an increased risk of thrombosis, especially in presence of atrial fibrillation and other triggering factors such as infections. Thrombosis in such patients should be suspected and promptly treated because of the high risk of early mortality.
- *Morganella morganii* is an emerging cause of severe infection that may be complicated by disseminated intravascular coagulation and cardiac thrombosis.

## Introduction

Disseminated intravascular coagulation (DIC) is a condition that encompasses both thrombosis and bleeding complications. The pro-thrombotic state in DIC derived from the activation of systemic coagulation and the derived consumption coagulopathy is responsible for the risk of haemorrhages. Disseminated intravascular coagulation is an acquired syndrome triggered by infectious and non-infectious causes. and it is rarely associated with heart failure (HF).^1,2^  *Morganella morganii* is an emerging pathogen, a potential cause of bloodstream infection with a 30-day mortality rate of ∼20%,^3,4^ but the association with DIC is not yet described. Here, we report a complex case of a patient with newly diagnosed HF and intra-cardiac thrombosis complicated by *M. morganii* infection and DIC.

## Summary figure

**Figure:**
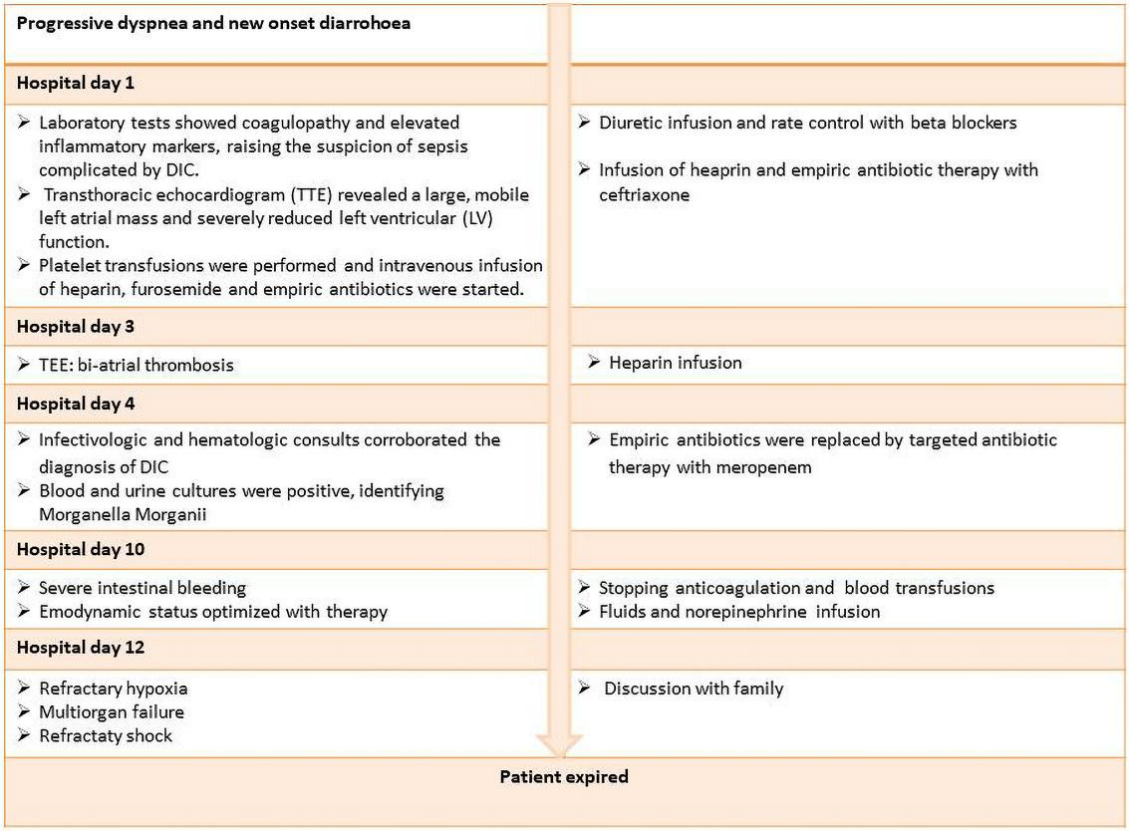


## Case presentation

A 69-year-old Caucasian man was admitted to the emergency department (ED) for progressive dyspnoea and new-onset diarrhoea. He has no significant illness in his previous medical history, except for hypertension and Type 2 diabetes. On admission, he was in orthopnaeic position with bilateral pleural effusion on chest X-ray. Physical examination revealed a Grade 2 systolic murmur on cardiac apex, leg swelling, and lung crackles. Twelve-lead electrocardiogram showed new-onset atrial fibrillation (AF) with inverted T waves in inferior and lateral leads. Initial laboratory tests showed an elevated N-terminal pro-brain natriuretic peptide (35 000 pg/mL) and stable and persistently elevated cardiac troponin (troponin I ∼200 ng/L), so he was diagnosed with acute HF complicated by new-onset AF.

In the ED, initial vital signs revealed AF with median heart rate of 65 b.p.m., tachypnoea (respiratory rate 21/min), hypoxaemia (pulse oxygen saturation: 89%), and fever recorded as 37.5°C. Blood pressure was 100/65 mmHg.

**Figure 1:**
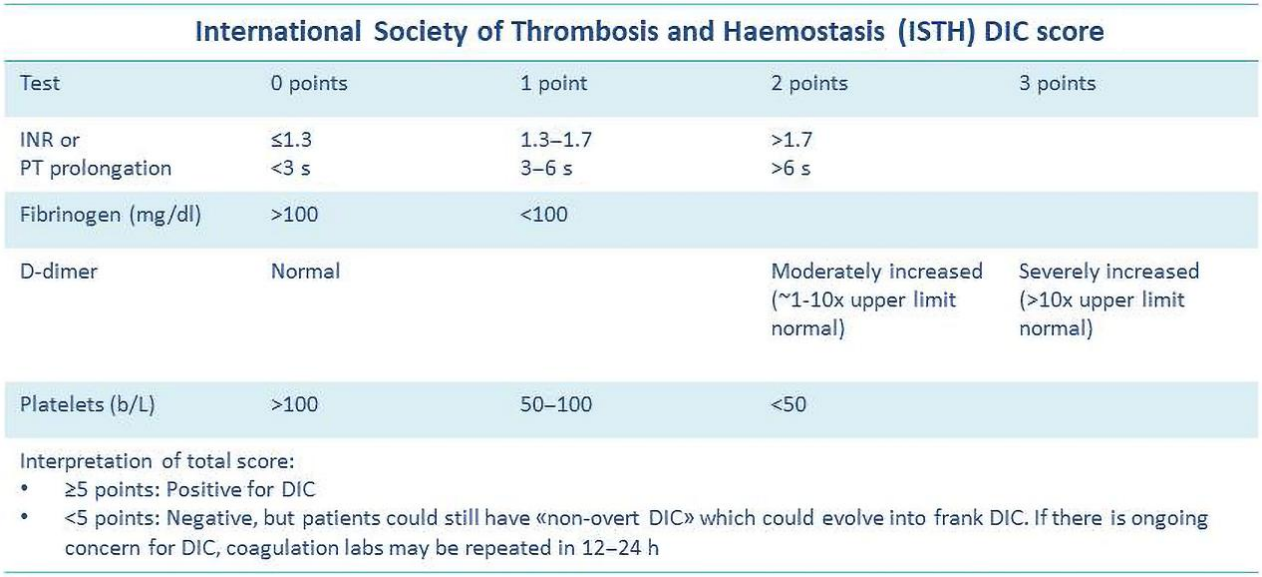
International Society on Thrombosis and Hemostasis criteria.

Further laboratory tests showed high white blood cell count (11 750/mm^3^, with 95.70% neutrophils), normal haemoglobin (13.60 g/dl), severe thrombocytopaenia (32 000/mm^3^), elevated C-reactive protein (166.66 mg/L), and procalcitonin (9.43 ng/mL), coagulopathy (International normalized ratio [INR] level of 2.04, fibrinogen level of 80 mg/dL, and D-dimer level of 13 380 ng/mL) in the presence of normal hepatic function, and acute kidney failure (serum creatinine of 2.06 mg/dL). Blood and urine cultures were positive, identifying *M. morganii*. In this context of urosepsis and coagulopathy, based on the clinical suspicion of DIC, the International Society on Thrombosis and Hemostasis (ISTH) criteria was performed (*Figure 1*), with a ISTH score of 6, diagnostic for overt DIC.

**Figure 2:**
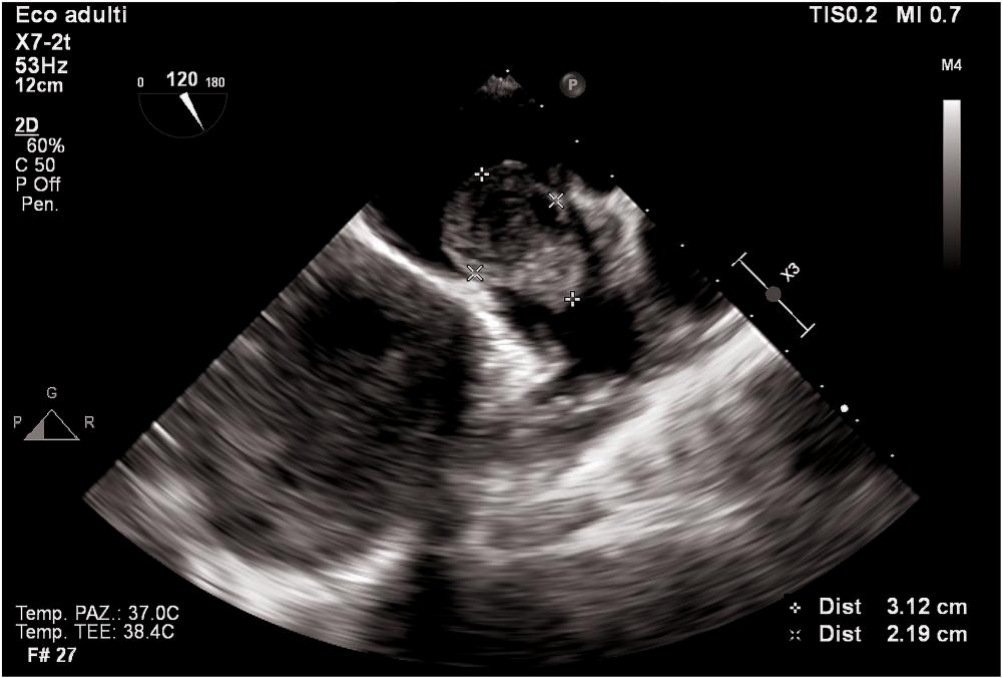
Transoesophageal echocardiogram mid-oesophageal view at 120°. The figure shows a huge mass in the left atrial appendage.

**Figure 3:**
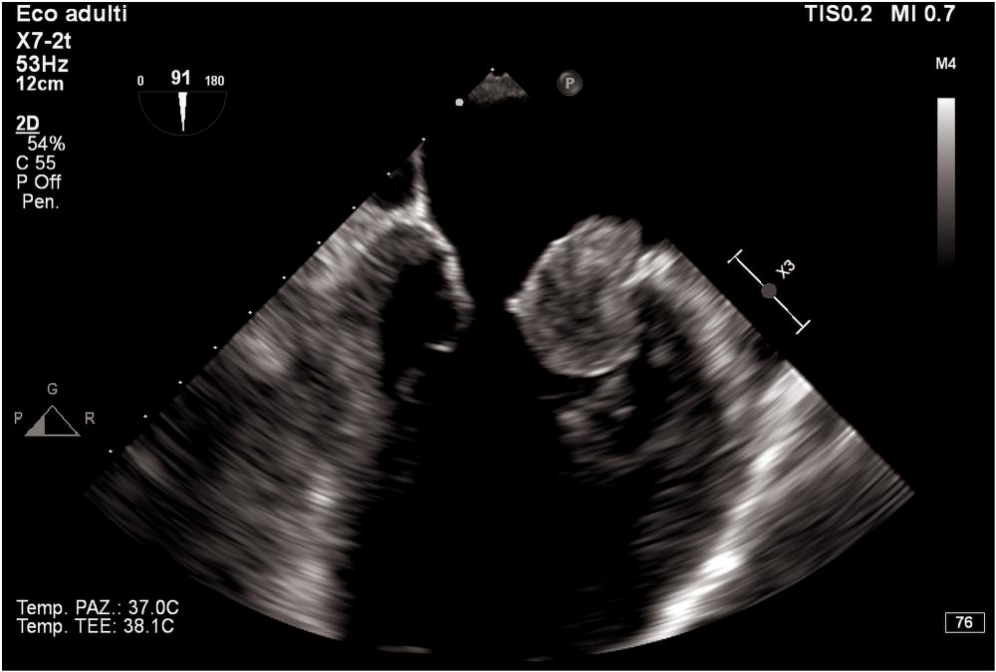
Transoesophageal echocardiogram mid-oesophageal view at 90° (two-chamber view). The figure shows the mass protruding in the left ventricle.

**Figure 4:**
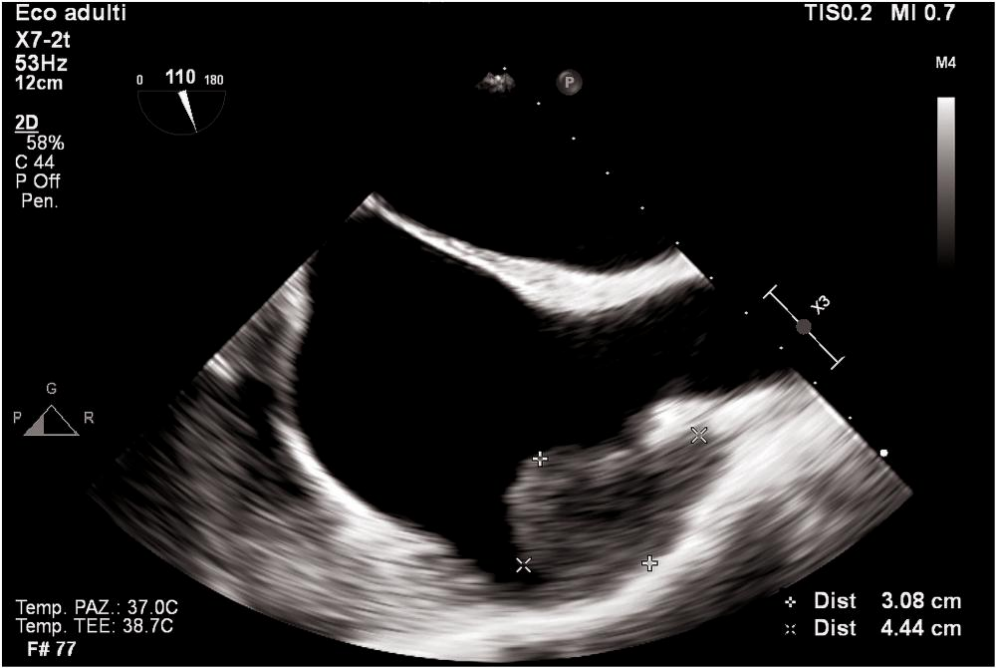
Transoesophageal echocardiogram mid-oesophageal view at 90° (bicaval view). The figure shows a mass in the right atrial appendage.

Transthoracic echocardiogram revealed left ventricular dilatation (end-diastolic volume was 155 mL), with reduced ejection fraction (EF 35%), global hypokinaesia (previous EF was 55% 1 year prior), and signs of elevated filling pressures (see Supplementary material online, *Video S1*). Both atria were enlarged (left atrium end-systolic volume was 90 mL). In the left atrium, a highly mobile rounded hyperechoic mass was observed. Tricuspid and mitral regurgitation were mild to moderate, and the systolic pulmonary artery pressure was 55 mmHg. Inferior vena cava was dilated without inspiratory collapse. No other clinically relevant valvulopathies, intra-cardiac masses, or vegetations were found. Three days later, transoesophageal echocardiogram (TEE) was performed trying to define the nature of the mass and showing a highly mobile mass within the left atrial appendage (LAA) and another mass in the right atrial appendage (RAA) (*Figures 2–4*; see Supplementary material online, *Video S2*).

**Figure 5:**
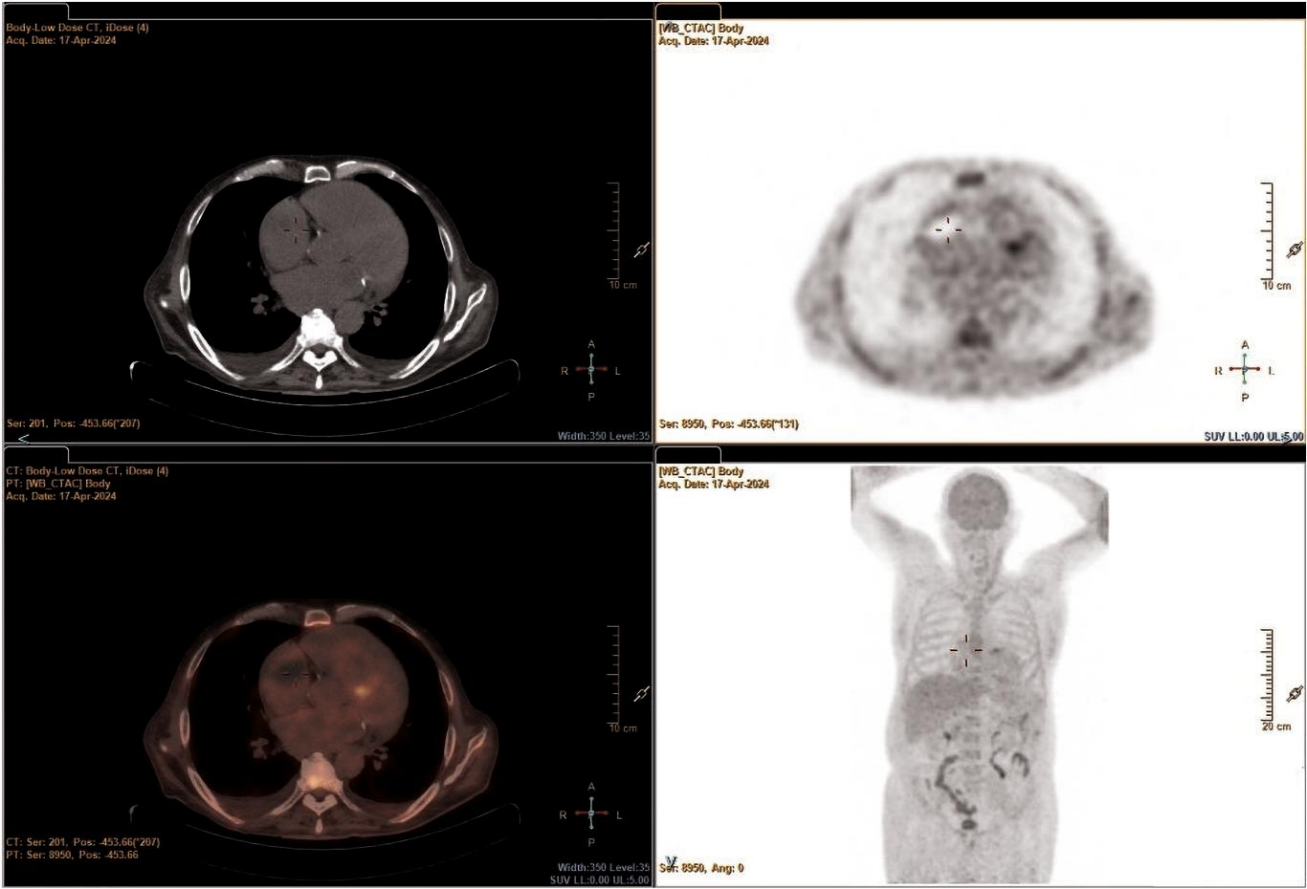
Positron emission tomography/computed tomography scan focused on the heart.

Differential diagnosis included thrombosis of the left and RAAs, myxoma, endocarditis, malignancy, or cardiac metastases. Tumour biomarkers were negative. Positron emission tomography/computed tomography scan (*Figure 5*) was performed and excluded both infective endocarditis and malignancies, defining the masses of thrombotic nature. Infectivological and haematologic consults corroborated the diagnosis of DIC.

After the admission, platelet transfusions were performed, with a rapid raise in platelet count. The patient started intravenous infusion of unfractionated heparin, monitored using activated partial thromboplastin time, furosemide 125 mg daily, and empiric antibiotic therapy with ceftriaxone and then replaced by targeted antibiotic therapy with meropenem for 4 days after the admission. Six days later, he had a severe intestinal bleeding from rectal varices with the necessity of blood transfusions and stopping anticoagulation. His haemodynamic status was initially optimized with fluids and norepinephrine infusion, but unfortunately it was complicated by liver and renal failure. At the end, after the initial haemorrhagic and septic shock, he developed also cardiogenic shock due to a superimposed septic cardiomyopathy unresponsive to medical and interventional treatments (extracorporeal membrane oxygenation).

## Discussion

Disseminated intravascular coagulation and HF are both associated with an altered haemostatic state^5,6^ and could be complicated by intra-cardiac thrombosis.^7,8^ Also AF adds to the thrombo-embolic risk profile.^9^

Disseminated intravascular coagulation is a pathologic syndrome characterized by intravascular fibrin formation in response to excessive blood protease activity. It is an uncommon but severe complication of an underlying disease and is most associated with sepsis, trauma, and malignancies. The treatment of DIC is controversial and must be highly individualized. The treatment principles are to remove the triggering process, stop the intravascular clotting process, and install component therapy.^6,8^

In patients with AF, the LAA may serve as site of thrombus formation due to the stasis and because of its shape and trabeculations. The incidence of thrombus formation in LAA during AF is ∼10–15%.^12^ Simultaneous bilateral atrial appendages thrombosis is an uncommon presentation in AF; in fact, RAA clot is rare with a frequency of <2%.^10–12^ This may be addressed to the anatomical features of RAA; in fact, it has a wider neck than LAA and a greater width-to-area ratio when compared with the LAA.^13–16^ Nevertheless, the lower reported number of RAA thrombi on TEE may be secondary to under-detected RAA thrombosis because of the inability to visualize the RAA on a TEE.

In the current literature, the associations between bilateral appendage thrombosis and DIC and between DIC and *M. morganii* infection are not yet described, although *M. morganii* is a potential cause of severe bloodstream infection. In this case, the exact mechanism of thrombus formation is unclear because multiple predisposing factors might be addressed: inflammatory storm, activation of coagulation cascade, and amplified immune response may create a vicious circle that could facilitate thrombosis. In this setting, DIC and HF mediated endothelial dysfunction,^5,17^ blood stasis in both atria was addressed to AF, and the increased blood viscosity might promote thrombosis in uncommon sites, such as RAA.

In our case, the appendage thrombosis and the need of anticoagulation therapy resulted in a severe intestinal bleeding that compromised the labile haemodynamic status of the patient.

## Conclusion

**Figure 6:**
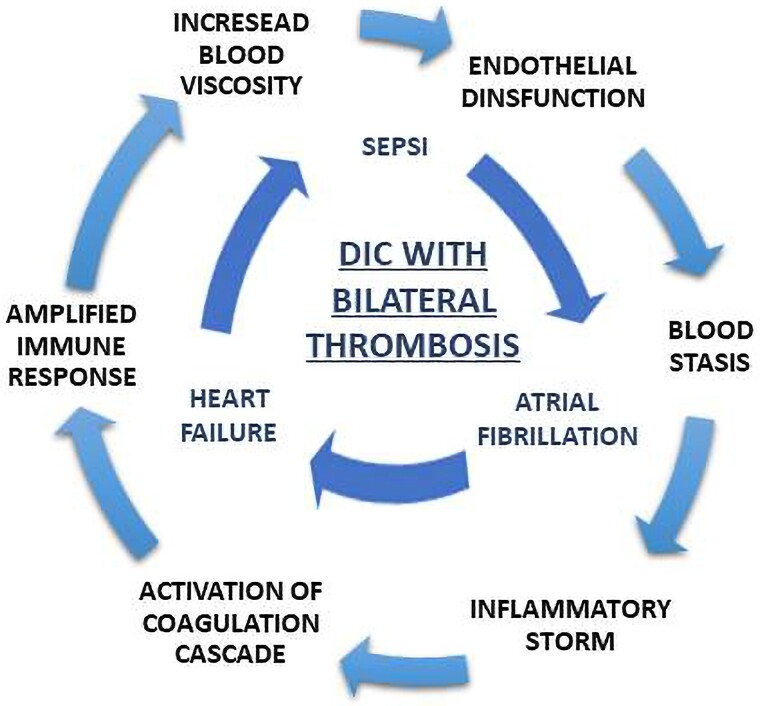
Central illustration.

This is the first reported case of concomitant cardiac thrombosis as a complication of DIC in the context of *M. morganii* infection. In our case, multiple predisposing factors such as HF, AF, and DIC may have facilitated RAA thrombosis, that is ‘per se’ an uncommon event in isolated AF (*Figure 6*). This highlights the need among clinicians for an increased awareness about this pathogen and the complications of its infection.

## Supplementary Material

ytaf043_Supplementary_Data

## Data Availability

The data underlying this article will be shared on reasonable request to the corresponding author.

## Lead author biography

Dr Mazzeo Pietro currently works at the Cardiology Unit of the San Carlo Hospital in Potenza. His field of interests are advanced echocardiographic imaging and heart failure.

## Supplementary material

**Consent:** Informed consent was obtained from a living relative for publication of this case report and any accompanying images in compliance with COPE guidelines. The relative has reviewed the content and agreed with the publication.

**Funding:** None declared.

## References

[1] Sarcon A, Liu X, Ton D, Haywood J, Hitchcock T. Advanced congestive heart failure associated with disseminated intravascular coagulopathy. J Investig Med High Impact Case Rep 2015;3:2324709615623298. 10.1177/2324709615623298 PMC4710112 26788528

[2] Belov D, Lyubarova R, Fein S, Torosoff M. Disseminated intravascular coagulation with congestive heart failure and left ventricular thrombus: a case report with literature review of 7 cases. Am J Case Rep 2015;16:53–56. 25637329 10.12659/AJCR.892380 PMC4315627

[3] Laupland KB, Paterson DL, Edwards F, Stewart AG, Harris PNA. *Morganella morganii*, an emerging cause of bloodstream infections. Microbiol Spectr 2022;10:e0056922. 35467403 10.1128/spectrum.00569-22 PMC9241912

[4] Erlanger D, Assous MV, Wiener-Well Y, Yinnon AM, Ben-Chetrit E. Clinical manifestations, risk factors and prognosis of patients with *Morganella morganii* sepsis. J Microbiol Immunol Infect 2019;52:443–448. 28919283 10.1016/j.jmii.2017.08.010

[5] Sbarouni E, Bradshaw A, Andreotti F, Tuddenham E, Oakley CM, Cleland JG. Relationship between hemostatic abnormalities and neuroendocrine activity in heart failure. Am Heart J 1994;127:607–612. 8122609 10.1016/0002-8703(94)90670-x

[6] Popescu NI, Lupu C, Lupu F. Disseminated intravascular coagulation and its immune mechanisms. Blood 2022;139:1973–1986. 34428280 10.1182/blood.2020007208 PMC8972096

[7] Heckman TA, Rosove MH. Massive left ventricular mural thrombosis with consumption coagulopathy in congestive heart failure. West J Med 1980;133:442–444. 7467300 PMC1272369

[8] Solomon SA, Cotton DWK, Preston FE, Ramsay LE. Severe disseminated intravascular coagulation associated with massive ventricular mural thrombus following acute myocardial infarction. Postgraduate Med J 1988;64:791–795. 10.1136/pgmj.64.756.791 PMC2428998 3255921

[9] Caldwell JC, Mamas MA, Neyses L, Garratt CJ. What are the thromboembolic risks of heart failure combined with chronic or paroxysmal AF? J Card Fail 2010;16:340–347. 20350702 10.1016/j.cardfail.2009.12.004

[10] Silverman DI, Manning WJ. Role of echocardiography in patients undergoing elective cardioversion of atrial fibrillation. Circulation 1998;98:479–486. 9714099 10.1161/01.cir.98.5.479

[11] Davila CD, Pandian NG. Simultaneous right and left atrial appendage thrombus in a patient with atrial fibrillation: a lesson to remember. Echocardiography 2015;32:1873–1875. 26332794 10.1111/echo.13044

[12] Klein AL, Grimm RA, Murray RD, Apperson-Hansen C, Asinger RW, Black IW, et al Use of transesophageal echocardiography to guide cardioversion in patients with atrial fibrillation. N Engl J Med 2001;344:1411–1420. 11346805 10.1056/NEJM200105103441901

[13] Subramaniam B, Riley MF, Panzica PJ, Manning WJ. Transesophageal echocardiographic assessment of right atrial appendage anatomy and function: comparison with the left atrial appendage and implications for local thrombus formation. J Am Soc Echocardiogr 2006;19:429–433. 16581482 10.1016/j.echo.2005.10.013

[14] De Divitiis M, Omran H, Rabahieh R, Rang B, Illien S, Schimpf R, et al Right atrial appendage thrombosis in atrial fibrillation: its frequency and its clinical predictors. Am J Cardiol 1999;84:1023–1028. 10569657 10.1016/s0002-9149(99)00492-0

[15] Davila CD, Pandian NG. Simultaneous right and left atrial appendage thrombus in a patient with atrial fibrillation: a lesson to remember. Echocardiography 2015;32:1873–1875. 26332794 10.1111/echo.13044

[16] Richardson AC, Omar M, Velarde G, Missov E, Percy R, Sattiraju S. Right atrial appendage thrombus in atrial fibrillation: a case report and review of the literature. J Investig Med High Impact Case Rep 2021;9:23247096211010048. 10.1177/23247096211010048 PMC8082980 33899523

[17] Okajima K, Sakamoto Y, Uchiba M. Heterogeneity in the incidence and clinical manifestations of disseminated intravascular coagulation: a study of 204 cases. Am J Hematol 2000;65:215–222. 11074538 10.1002/1096-8652(200011)65:3<215::aid-ajh7>3.0.co;2-7

