# Supplementary material for: Stuck between a rock and a hard place: heart failure with bilateral atrial appendage thrombi and disseminated intravascular coagulation—a case report
Source: Eur Heart J Case Rep. 2025 Feb 6;9(2):ytaf043. doi: 10.1093/ehjcr/ytaf043 (PMC11850653; doi:10.1093/ehjcr/ytaf043)
Supplement: ytaf043_Supplementary_Data [file ytaf043_supplementary_data.zip › Supplementary Captions.docx]

**Video 1** Transthoracic echocardiogram showing LV function

**Video 2** Transesophageal echocardiogram showing LAA and RAA masses
